# The climate changes promoted the chloroplast genomic evolution of *Dendrobium* orchids among multiple photosynthetic pathways

**DOI:** 10.1186/s12870-023-04186-y

**Published:** 2023-04-10

**Authors:** Qiqian Xue, Jiapeng Yang, Wenhui Yu, Hongman Wang, Zhenyu Hou, Chao Li, Qingyun Xue, Wei Liu, Xiaoyu Ding, Zhitao Niu

**Affiliations:** 1 College of Life Sciences, Nanjing Normal University, Nanjing, 210023 China; 2 Jiangsu Provincial Engineering Research Center for Technical Industrialization for Dendrobiums, Nanjing, 210023 China

**Keywords:** *Dendrobium*, Chloroplast genome, CAM evolution, InDel, Positive selection, Climatic analysis

## Abstract

*Dendrobium* orchids have multiple photosynthetic pathways, which can be used as a model system for studying the evolution of crassulacean acid metabolism (CAM). In this study, based on the results of the net photosynthetic rates (P_n_), we classified *Dendrobium* species into three photosynthetic pathways, then employed and compared their chloroplast genomes. The *Dendrobium* chloroplast genomes have typical quartile structures, ranging from 150,841–153,038 bp. The apparent differences in GC content, sequence variability, and IR junctions of SSC/IR_B_ junctions (J_SB_s) were measured within chloroplast genomes among different photosynthetic pathways. The phylogenetic analysis has revealed multiple independent CAM origins among the selected *Dendrobium* species. After counting insertions and deletions (InDels), we found that the occurrence rates and distribution densities among different photosynthetic pathways were inconsistent. Moreover, the evolution patterns of chloroplast genes in *Dendrobium* among three photosynthetic pathways were also diversified. Considering the diversified genome structure variations and the evolution patterns of protein-coding genes among *Dendrobium* species, we proposed that the evolution of the chloroplast genomes was disproportional among different photosynthetic pathways. Furthermore, climatic correlation revealed that temperature and precipitation have influenced the distribution among different photosynthetic pathways and promoted the foundation of CAM pathway in *Dendrobium* orchids. Based on our study, we provided not only new insights into the CAM evolution of *Dendrobium* but also provided beneficial genetic data resources for the further systematical study of *Dendrobium*.

**Supplementary Information:**

The online version contains supplementary material available at 10.1186/s12870-023-04186-y.

## Introduction

Crassulacean acid metabolism (CAM) is a photosynthetic pathway that has arisen convergently in many plant lineages, especially for the species that live in CO_2_ and water-limited environments, such as some aquatic habitats, hot semiarid areas, and tropical forests [1, 2]. Recent studies have revealed the multiple independent origins of the CAM pathway, which have occurred in at least 343 genera across 35 plant families, accounting for 6 percent of vascular plants [3–5]. For example, in the Bromeliaceae family, CAM photosynthesis evolved at least three times due to their epiphytic habitats [6], while because of climate changes, three distinct evolutions of CAM pathways have been detected in the Agavoideae family [7]. Additionally, it has indicated that most epiphyte plants evolved with the CAM pathway [3], e.g., bromeliads [8]; pteridophytes [4]; and especially orchid species [9, 10].

The Orchidaceae family, one of the largest families, contains more than 28,000 species, which are widely distributed in East Asia, South-East Asia, and Oceania [11]. The unique habitats have forced the adaptive radiation of orchid species, resulting in their diversified characters, e.g., the epiphytic habits, deceit pollination, and the presence of the CAM pathway. Previous studies have shown that CAM pathway has evolved independently among orchid species. Firstly, CAM photosynthesis evolved independently among different orchid genera, as shown in Bone et al. [12], where CAM pathway has evolved ten and four times independently among Neotropical and Eulophiinae orchids, respectively. Secondly, CAM photosynthesis has also evolved independently within the orchid genus. For example, in the genus *Dendrobium*, CAM pathway has arisen at least eight times independently [13]. Thus, orchid species have shown a diversified evolution of their photosynthetic pathway.

Chloroplast, the main reaction center of photosynthesis, is the most crucial organelle for plant growth and development. Recently, research on the orchid chloroplast genome have revealed that: (i) independent absence of *ndh* genes in different orchid lineage, e.g. *ndh A*, *E*, *F*,* I* and *K* lacked sequence in *Erycina pusilla* [14], while *ndh A*, *F*, and *H* genes were utterly absent in *Phalaenopsis Aphrodite* [15]. (ii) the substitution rates among protein-coding genes vary in photosynthetic orchid chloroplast genomes, e.g., *psbC* elucidated the highest synonymous substitution rates (ds) value in *Apostasia,* but the lowest in *Phalaenopsis*, while *rpl36* contained the lowest ds value in the *Apostasia,* but highest in *Phalaenopsis* [16]. (iii) the evolution rates of the non-coding regions were diversified. For instance, *Cymbidium*, *Phalaenopsis* and *Apostasia* showed inconsistent patterns in the top-10 mutational hotspots among various orchid genera [17–19]. Although numerous studies have demonstrated the diverse evolution of chloroplast genomes in orchids, there remains a shortage of information about the relationship between the evolution of the CAM pathway and the disproportional variation among orchid chloroplast genomes.

*Dendrobium*, one of the essential genera of orchids, comprises roughly 120 species in China and has unique habitats with a wide geographical distribution, from Asian regions to New Zealand, and a large altitude distribution. It attaches to tree trunk rocks between 200 and 1800 m. The unique habitats have led to various photosynthetic pathways, such as C_3_ pathway, facultative CAM pathway, and CAM pathway, among different species. For example, *D. officinale* has been shown to be a C_3_-CAM plant [9], *D. primulinum* has been indicated to be a CAM plant [20], while *D. baileyi* exhibited certain characteristics of a C_3_ plant [21]. Consequently, the diversified photosynthetic pathways of *Dendrobium* species could be utilized as a model system to research the evolution of CAM. In this study, we analyze eleven *Dendrobium* chloroplast genomes, including nine newly sequenced and two previously published genome sequences, to address three questions: (a) Could the comparative plastomic approaches screen available structural differences? (b) If so, are these differences related to different photosynthetic pathways in *Dendrobium* species? (c) Are the photosynthetic pathways and geographical distribution in *Dendrobium* correlated, or does the geographical distribution promote the evolution of photosynthetic pathways in *Dendrobium*? To address these problems, we compared the plastomic structures among *Dendrobium* chloroplast genomes and evaluated the evolutional rates of protein-coding genes. Moreover, based on climatic analysis and selection forces, we studied the relationship between geographical distribution and photosynthetic pathways in *Dendrobium*. The integrative summary of findings in this research could provide further insights into the climatic factors and chloroplast features enabling CAM evolution in *Dendrobium*.

## Materials and methods

### Plant materials and DNA extraction

In this study, eleven *Dendrobium* orchids (*Dendrobium primulinum* Lindl. (voucher specimen: Xue202201), *Dendrobium longicornu* Lindl. (voucher specimen: Xue202202), *Dendrobium terminale* Par. et Rchb. F. (voucher specimen: Xue202203), *Dendrobium chrysotoxum* Lindl. (voucher specimen: Xue202204), *Dendrobium nobile* Lindl. (voucher specimen: Xue202205), *Dendrobium acinaciforme* Roxb. (voucher specimen: Xue202206), *Dendrobium thyrsiflorum* Rchb. (voucher specimen: Xue202207), *Dendrobium officinale* Kimura et Migo (voucher specimen: Xue202208), *Dendrobium lindleyi* Stendel. (voucher specimen: Xue202209), *Dendrobium chrysanthum* Lindl. (voucher specimen: Xue202210), *Dendrobium hercoglossum* Rchb. f. (voucher specimen: Xue202211)) were stored in College of Life Sciences, Nanjing Normal University, Nanjing, China. Utilizing Dneasy Plant Mini Kits (QIAGEN, Germany), the total genomic DNA of individuals was extracted from 2 g of healthy leaves. The A260/280 ratio of the DNA samples utilized for sequencing was between 1.8 and 2.0, while the A260/230 ratio should be larger than 1.7. In addition, the DNA content should be higher than 300 ng/μL of each DNA sample.

### Determination of the net photosynthetic rate

After 45 days of planting in the greenhouse of College of Life Sciences, Nanjing Normal University, we measured the net photosynthetic rates (P_n_) of 11 *Dendrobium* orchids (*Dendrobium primulinum*, *Dendrobium longicornu*, *Dendrobium terminale*, *Dendrobium chrysotoxum*, *Dendrobium nobile*, *Dendrobium acinaciforme*, *Dendrobium thyrsiflorum*, *Dendrobium officinale*, *Dendrobium lindleyi*, *Dendrobium chrysanthum*, *Dendrobium hercoglossum*). During the experiment in the greenhouse, the mean temperature was 25 °C; the mean atmospheric relative humidity was 80%; and the mean light intensity was 620 μmol/m^2^/s. A portable photosynthesis system (CIRAS-3, PP SYSTEMS, American) was used to measure P_n_ of 11 *Dendrobium* species. To assess diurnal variation in photosynthesis, measurements were taken at different periods every 24 h for eight days under natural light conditions. All measurements were conducted on the top second leaf, comprising 10 biological replicates per sample.

### DNA sequencing, assembly, and annotation

The Illumina Hiseq4000 platform was used to sequence the whole genomic DNA of nine *Dendrobium* orchids. With 150 bp paired-end reads for individuals, almost 27.78 Gb of raw data were generated. The fragments with coverage less than 50 × were eliminated and filtered paired-end reads were assembled on CLC Genomics Workbench v8.5.1 (CLC Bio, Aarhus, Denmark) with reference *Dendrobium officinale* Kimura et Migo (NC_024019). To annotate the assembled genomes, DOGMA v1.2 and tRNAscan-SE v1.21 [22, 23] were used. By using BLAST and multiple sequence alignment, the annotated genes were corrected.

### Comparative analysis of chloroplast genomes

After extracting the information about the gene location, it was combined with the information about IR/SC junctions. The GC contents of 11 *Dendrobium* species were also investigated. Meanwhile, the nine *Dendrobium* chloroplast genomes, which were newly sequenced, with two published *Dendrobium* were compared using online mVISTA on LAGAN model with reference *Bulbophyllum inconspicuum* Maxim. [24]. The IR/SC junctions map was generated using 11 *Dendrobium* orchids with *B. inconspicuum* as a reference, referring to the drawing approach of Zhu et al. [25] of four junctions.

### Phylogenetic relationship and divergence time estimation

On the basis of 31 complete chloroplast genomes, comprising 13 *Dendrobium* plants and other Orchidaceae species, phylogenetic relationships were examined. (Supplementary Table 2). The chloroplast genome sequences of the 31 angiosperms were aligned using MAFFT 7.221 [26]. The gaps were deleted by Gblocks v.0.91b [27]. The best base substitution model determined by Modeltest 3.7 according to the AIC (Akaike information criterion) rule was GTR + I + Γ [28]. Using RAxML v.7.4.2 [29] and MrBayes 3.2.7 [30] separately, the Maximum Likelihood (ML) and Bayesian inference (BI) phylogenetic trees were created. We estimated divergence times by BEAST2 [31].

Time calibrations were conducted with the following restrictions: (1) A root age of 82.5 million years ago (mya) was selected (prior distribution: normal, mean: 82.5, sd: 5) [32]. (2) The separation between the Asian and Australian clades in *Dendrobium*, the *Dendrobium* Crown age, was determined to have occurred 23.2 mya based on the fossil record (prior distribution: exponential, offset: 23.2, mean: 8) [33, 34]. Convergence was tested using three independent MCMC, each containing 100,000,000 generations. Three separate runs were merged with LogCombiner to discard the top 10% of unreliable data.

### Structural variation analysis of *Dendrobium* chloroplast genomes

The chloroplast genomes of 11 *Dendrobium* species were aligned using MAFFT 7.220 [26] with *B. inconspicuum* as a reference. The gaps at both ends were deleted. The insertions/deletions (InDels) of every *Dendrobium* chloroplast genome were measured, with *B. inconspicuum* as a reference. To determine the occurrence rates of InDels, the InDels of 11 *Dendrobium* chloroplast genomes were collected.

### Substitution rates and positive selection analysis

The chloroplast genomes of 11 *Dendrobium* species were evaluated synonymous (ds) and non-synonymous (dn) substitution rates by the CodeML program of PAML (version 4.4) with reference *B. inconspicuum* [35, 36]. Then we examined the molecular evolution of 68 protein-coding genes from 11 *Dendrobium* species with reference *B. inconspicuum*. The value of dn/ds, dn and ds was also assessed by the CodeML program. To prevent the misestimating of dn/ds, the 35 genes with high ds values were eliminated. To determine the significance of genes among multiple photosynthetic pathways, we screened *Dendrobium* chloroplast protein-coding genes with various dn/ds values (Kruskal–Wallis test for Independent Samples). Then, we used Codeml in PAML to perform the branch model analysis to look for adaptively evolving genes in 11 *Dendrobium* orchids. At a threshold of *P* < 0.05, the likelihood ratio test (LRT) with a χ^2^ distribution was employed to identify whether models were significantly varied from the null model [37].

### Climatic analyses

After obtaining reliable collection information of the 11 species from the database, 13 records per species were obtained. From the official WorldClim website(worldclim.org), 19 WorldClim (v. 2.1) bioclimatic layers (Supplementary Table 5) were acquired. These climatic layers contain annual trends (e.g., mean annual temperature, annual precipitation), seasonality (e.g., annual range in temperature and precipitation), and harsh or constricting abiotic factors (e.g., temperature of the coldest and warmest month, and precipitation of the wet and dry quarters). The climatic layers were constructed using data from records spanning the years 1970 to 2000, with a spatial resolution of 1 km^2^. With DIVA-GIS v.7.5 as the ecological resource, 19 environmental factors were retrieved from the bioclimatic layer of each locality (13 localities per species × 11 species × 19 environmental factors) [38]. Principal component analysis (PCA) was run with R (v.4.0.3) by using the bioclimatic dataset of localities to ensure the connection between photosynthetic pathways and climatic patterns in *Dendrobium*. PCA was performed in R (v.4.0.3) utilizing the bioclimatic record of locations to investigate the association between photosynthetic pathways and climatic variation in *Dendrobium*.

## Results

### Determination of photosynthetic pathways in *Dendrobium* species

The net photosynthetic rate (P_n_) of 11 *Dendrobium* species was analyzed in this study. Based on the results, the 11 *Dendrobium* species could be classified into three different categories (Supplementary Fig. 1). *D. chrysotoxum*, *D. longicornu*, *D. chrysanthum*, *D. thyrsiflorum*, and *D. lindleyi* belong to C_3_ plants because their P_n_ expanded zero during daytime but were lower than that of the night. Meanwhile, *D. primulinum*, *D. terminale* and *D. acinaciforme* have the opposite trends of P_n_, which identifies them as CAM plants. Finally, *D. officinale*, *D. nobile*, and *D. hercoglossum* were identified as C_3_-CAM plants according to their P_n_ values, which exceeded zero on both day and night.

### Chloroplast genome features of *Dendrobium* species

**Fig. 1:**
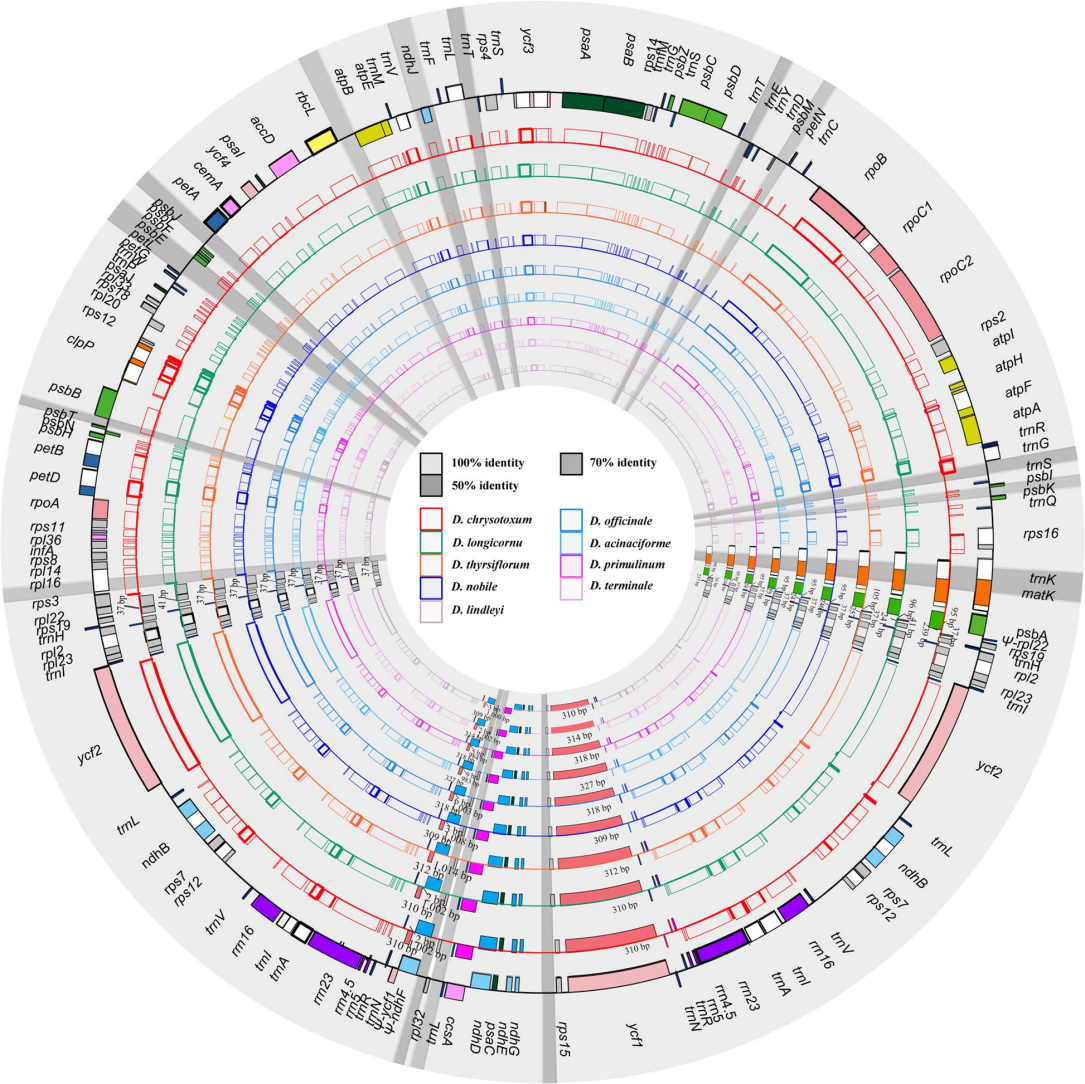
The plot shows the basic characteristics of the nine chloroplast genomes acquired in this study. The outer circle shows gene placement and annotation across the genome. Genes are represented in different colors. Positive and negative gene orientations are shown as outer and inner circles. Inner circles represent nine newly sequenced *Dendrobium* chloroplast genomes. The high-identity areas are highlighted in light grey for chloroplast genome sequence variability (100%). *Dendrobium* species are displayed using distinct colors, which mark the length of four IR/SC junctions

To date, more than 30 *Dendrobium* chloroplast genomes have been sequenced; however, their photosynthetic pathway remains unclear. Thus, we selected only eleven species for our comparative chloroplast genomic studies based on the results of the photosynthetic experiment (Supplementary Fig. 1). We summarized the genomic features of eleven *Dendrobium* chloroplast genomes, including nine newly sequenced and two published *Dendrobium*, were summarized (Fig. 1). According to Table 1, the GC contents of the *Dendrobium* chloroplast genomes ranged from 37.47% to 37.61%, with sizes ranging from 150,841to 153,038 bp. The sizes of LSC, SSC and IR regions were 83,932 bp to 85,068 bp, 14,023 bp to 14,523 bp and 26,291 bp to 27,030 bp, respectively. The GC contents varied slightly among the eleven chloroplast genomes in LSC (35.01 -35.20%), SSC (30.21–30.92%) and IR (43.17–43.39%) regions.

**Table 1:** The characteristics of *Dendrobium* chloroplast genomes

No	Species	Accession	Reference	Chloroplast genome	LSC	SSC	IR	GC content (%)			
		no		length (bp)	length (bp)	length (bp)	length (bp)	Total	LSC	SSC	IR
1	*D. primulinum*	LC635345	This study	151,603	84,849	14,157	26,300	37.51	35.08	30.21	43.38
2	*D. hercoglossum*	LC490400	Li et al. [39]	152,136	84,988	14,476	26,336	37.49	35.08	30.33	43.35
3	*D. longicornu*	LC635347	This study	151,789	84,780	14,418	26,297	37.5	35.04	30.66	43.36
4	*D. terminale*	LC635346	This study	151,490	84,451	14,432	26,305	37.54	35.11	30.81	43.28
5	*D. chrysotoxum*	LC635348	This study	151,895	84,853	14,463	26,291	37.59	35.18	30.71	43.38
6	*D. nobile*	LC636120	This study	152,089	85,018	14,464	26,305	37.52	35.06	30.62	43.39
7	*D. lindleyi*	LC636121	This study	151,943	84,959	14,409	26,291	37.55	35.11	30.65	43.39
8	*D. acinaciforme*	LC636124	This study	150,841	83,932	14,278	26,317	37.61	35.2	30.92	43.27
9	*D. thyrsiflorum*	LC636122	This study	151,926	84,836	14,425	26,334	37.48	35.01	30.7	43.33
10	*D. chrysanthum*	LC490683	Li et al. [39]	153,038	84,955	14,023	27,030	37.49	35.06	30.34	43.17
11	*D. officinale*	LC636123	This study	152,208	85,068	14,523	26,310	37.47	35.05	30.32	43.36

The levels of chloroplast genome sequence variability were evaluated among eleven *Dendrobium* species using mVISTA, with *Bulbophyllum Inconspicuum* as a reference (Fig. 1). The comparison results showed: (i) variable genome sequence of non-coding regions than coding regions; (ii) higher variability of SC regions than IR regions. These results showed the same trend as the previous studies [16, 19].

### Comparison of sequences flanking IR/SC junctions

The sequences flanking IR/SC boundaries among *Dendrobium* were compared. As shown in Supplementary Fig. 2, the *Dendrobium* IR/SC boundaries were highly conserved. The pseudogene fragment *Ψycf1* (309 to 327 bp) in the SSC/IR_B_ junctions (J_SB_s) was caused by the SSC/IR_A_ junctions (J_SA_s), which were situated at the 5’ end of *ycf1*. Meanwhile, the LSC/IR_B_ junctions (J_LB_s) were situated in *rpl22*, resulting in *Ψrpl22* (37 to 41 bp) located in the LSC/IR_A_ junctions (J_LA_s). Notably, the IR_B_ gradually expands to *ΨndhF*. The main difference between these three categories of chloroplast genomes exists in J_SB_s, which can be further classified into two types: (a) an overlap of *Ψycf1* and *ΨndhF* by 2–18 bp in CAM and part of C_3_ categories (*D. chrysanthum* and *D. thyrsiflorum*); (b)a gap between *Ψycf1* and *ΨndhF* by 0–3 bp in C_3_-CAM and the rest of C_3_ categories (*D. chrysotoxum*, *D. longicornu*, *D. lindleyi*). These findings suggested that the evolution of IR/SC boundaries among the three categories was diverse.

### Divergence time estimation

**Fig. 2:**
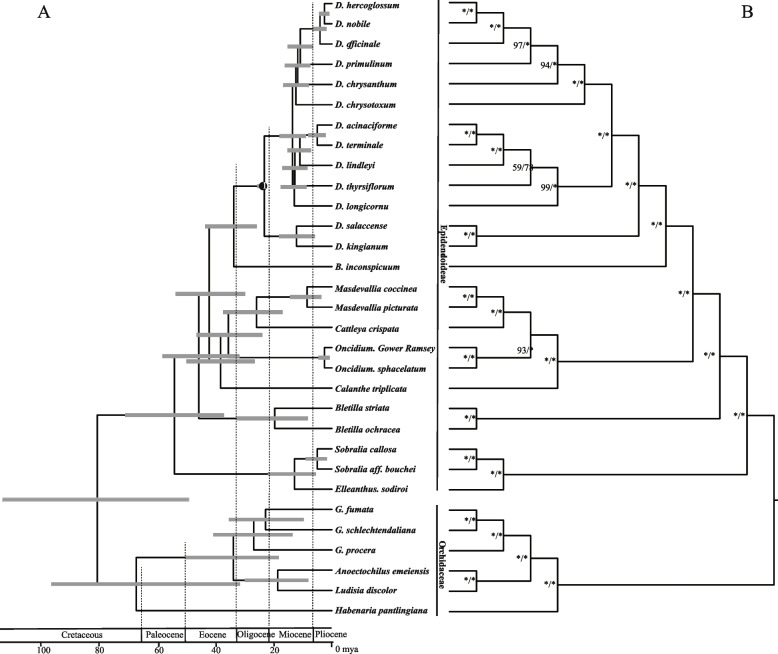
Chronogram and Phylogenetic trees of 31 species. **A** Molecular dating results of 31 angiosperms by BEAST2; **B** ML tree topology with ML and BI bootstrap values. The first one represents ML bootstrap value and the second one represents BI bootstrap value. * represents 100 bootstrap value

To estimate the divergence times of *Dendrobium* species, we reconstructed a phylogenetic tree using 31 complete angiosperm chloroplast genomes, including 13 *Dendrobium* species (Supplementary Table 1). The phylogenetic trees revealed that the orchids have a monophyletic relationship with strong support (bootstrap values ML/BI = 100/100, Fig. 2B). Furthermore, the *Dendrobium* was monophyletic with 100/100 bootstrap values and was sister to *Bulbophyllum*. To estimate the divergence times of *Dendrobium* species, we also constructed a BEAST tree. Meanwhile, the topologies of the BEAST tree were similar to ML and BI trees (Fig. 2A). Then, we estimated the divergence times for each node. As expected, the 13 *Dendrobium* species were separated into Asian and Australian clades at 23.86 (23.20–26.25) mya. Within the Asian clade, the photosynthetic pathways have evolved independently among different *Dendrobium* species, e.g., CAM pathway has evolved from C_3_ pathway twice, with the first arising at 12.07 (7.68–16.54) mya and the second diverged at 11.31 (7.41–15.61) mya independently.

### Structural variation analysis of chloroplast genomes

**Fig. 3:**
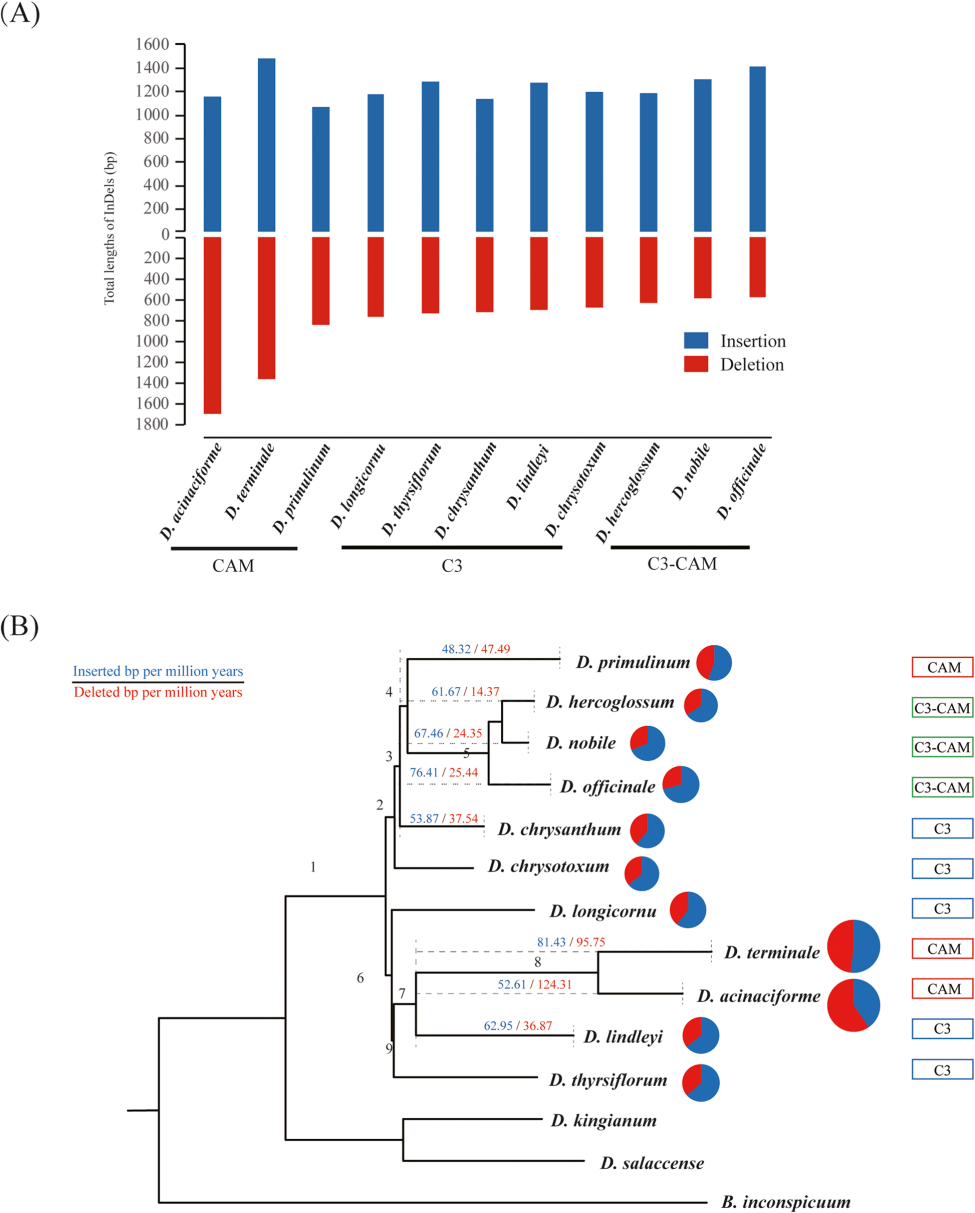
The occurrence rates of chloroplast genomic variation during speciation in *Dendrobium*. **A** Overall length of InDels for different lineages of *Dendrobium* with *B. inconspicuum* as reference; **B** Accumulation rates of InDels lengths every million years along branches of the *Dendrobium* phylogeny. Pies of 11 *Dendrobium* species are scaled proportionally to InDel lengths. Divergence times (myr) of 1–9 branches are estimated by Yang et al. [32]. Major branches of insertion and deletion lengths every million years are shown

The insertions and deletions (InDels) among 11 *Dendrobium* chloroplast genomes were identified with *B. Inconspicuum* as an outgroup. In *Dendrobium* chloroplast genomes, had a higher proportion of deletions (575 to 1,688 bp) than insertions (1,063 to 1,474 bp) (Fig. 3A). Meanwhile, the distribution densities of InDels in the SSC region were higher than in LSC and IR regions in *Dendrobium*, indicating that the InDels distribution differed among chloroplast genomes (Supplementary Fig. 3). In addition, the occurrence of InDels was variable among C_3_, CAM and C_3_-CAM *Dendrobium* orchids. For example, (i) the deletion lengths differed among the three categories. The total deletion length of the CAM category (841 to 1,688 bp) was higher than the C_3_-CAM category (575 to 623 bp) and C_3_ category (671 to 760 bp) (Fig. 3A). (ii) the occurrence rates of InDels among three categories were inconsistent (Fig. 3B). The InDels occurrence rates in CAM category (88.26 and 93.27 bp/myr) were partially higher than those in C_3_ category (86.53 and 51.48 bp/myr) and C_3_-CAM category (92.86 and 42.73 bp/myr). These findings demonstrated that InDels evolved diversely among three categories.

### Evolutional analysis of protein-coding genes

**Fig. 4:**
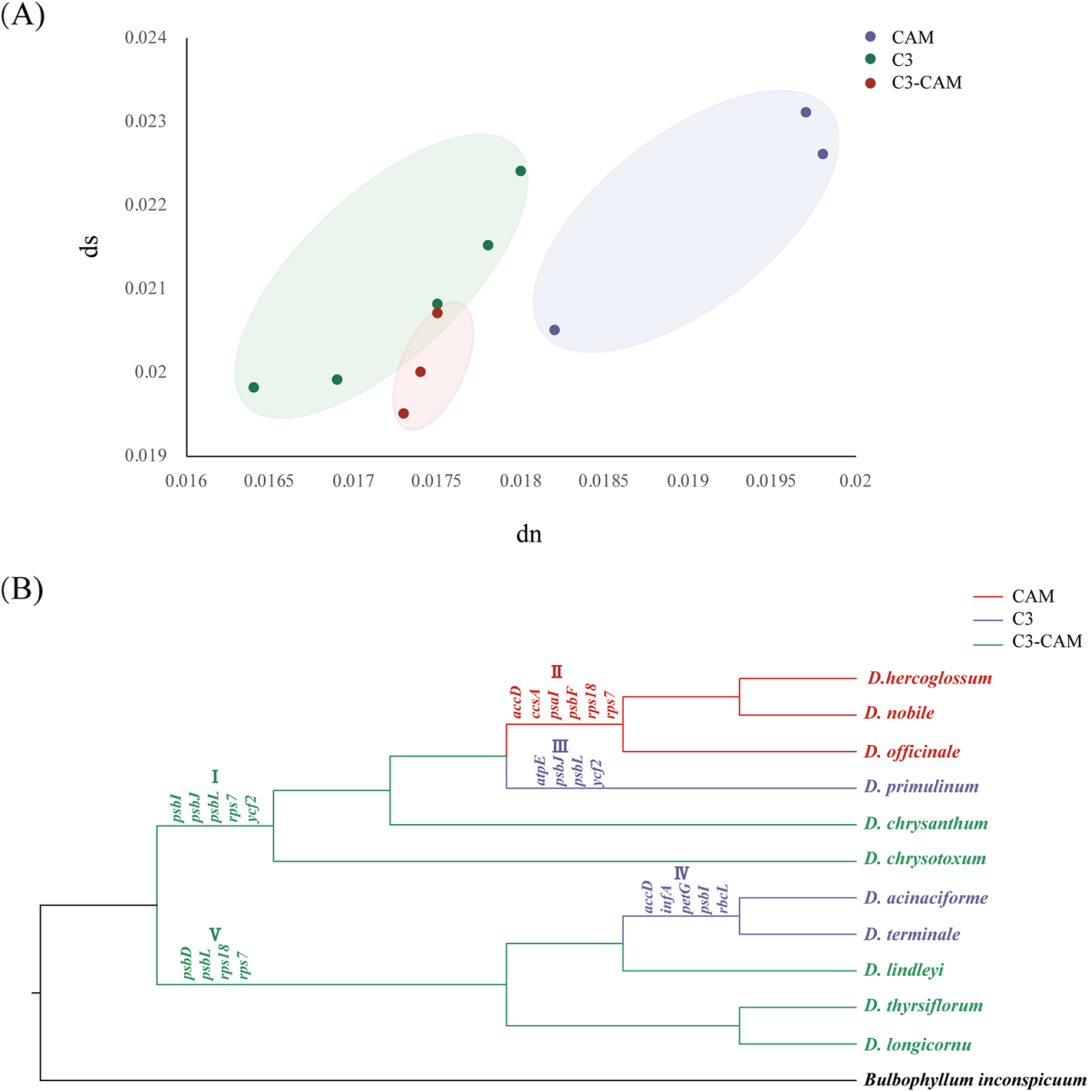
The results of evolutional analysis of protein-coding genes in *Dendrobium*. **A** Comparison of non-synonymous (dn) and synonymous (ds) substitution rates among three photosynthetic pathways (C_3_, CAM and C_3_-CAM). The substitutions rates were calculated for the whole chloroplast genome with *B. inconspicuum* as reference. Of note, the chloroplast genomes of *Dendrobium* among three photosynthetic pathways revealed various substitution rates in their protein-coding sequences; **B** ML tree with adaptive selection genes in different branches among *Dendrobium* species, respectively. Different colors are used to mark different branches

Among the chloroplast genomes of 11 *Dendrobium* orchids, the evaluated synonymous (ds) and non-synonymous (dn) substitution rates were 0.0164–0.0198 and 0.0195–0.0231, respectively. The chloroplast genomes of *Dendrobium* among three photosynthetic pathways (CAM, C_3_ and C_3_-CAM) exhibited various substitution rates, with dn ranging from 0.0164–0.0198 and ds ranging from 0.0195–0.0231, while CAM (dn: 0.0205–0.0226, ds: 0.0205–0.0231) exhibited notably higher substitution rates than those of the other photosynthetic species. The ds and dn of every protein-coding gene were evaluated using CodeML, with *B. Inconspicuum* as reference. The values of ds (0.0091–0.0909) were higher than those of dn (0.0019–0.8205) in all branches, indicating that the substitution rates of protein-coding genes were diversified. In addition, the values of dn and ds differed among C_3_, CAM and C_3_-CAM *Dendrobium* orchids. Most of the dn/ds values among the multiple photosynthetic pathways varied (Fig. 4A), comprising several remarkably different protein-coding genes (Supplementary Table 2). For instance, the dn rates of the genes of *clpP*, *matK* and *ycf1* were highest in the CAM category but lowest in the C_3_ category (Supplementary Table 2). These genes, which functioned in self-replication and photosynthesis, were typically located in LSC regions (Supplementary Table 3). These findings revealed that the evolution of some protein-coding genes was inconsistent among multiple photosynthetic pathways.

### Positively selected chloroplast genes

The Branch model of CodeML was used to examine the potential role of positive selection in promoting the evolution of protein-coding genes among distinct photosynthetic pathways (C_3_, CAM and C_3_-CAM). Comparative analysis revealed that 24 genes were under various selection pressure. For example, there were five genes (*psbI, psbJ, psbL, rps7* and *ycf2*) were discovered in branch I; six genes (*accD, ccsA, psaI, psbF, rps18* and *rps7*) were found in branch II; four genes (*atpE, psbJ, psbL* and *ycf2*) were found in branch III; five genes (*accD, infA, petG, psbI* and *rbcL*) were found in branch IV; and four genes (*psbD, psbL, rps18* and *rps7*) were found in branch V (Fig. 4B). These genes were mostly related to the main components of Photosystems I and II (Supplementary Table 4), indicating that the adaptive evolution of genes was correlated with photosynthetic pathways.

### Climatic analyses

**Fig. 5:**
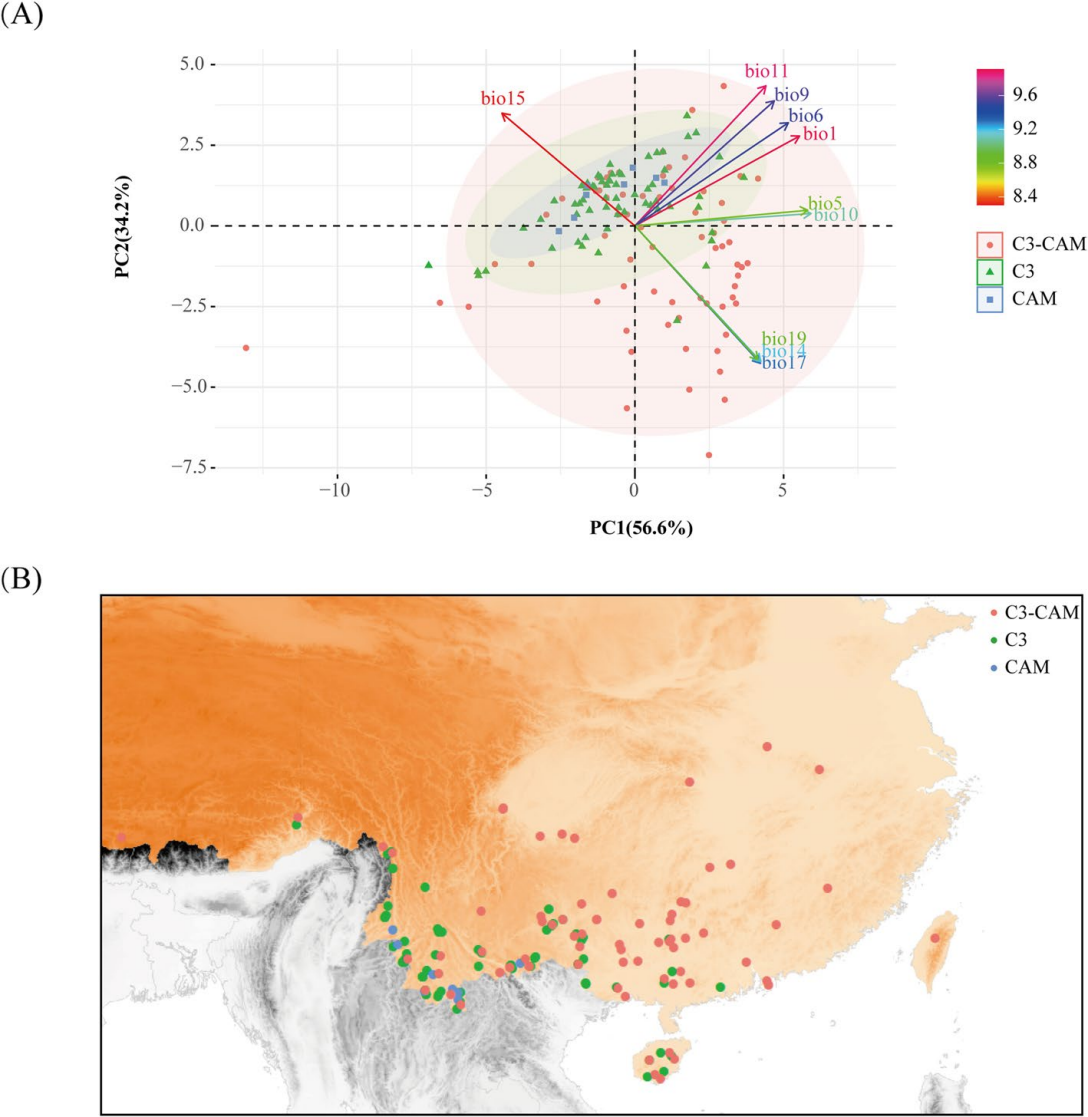
Climatic analysis of 11 *Dendrobium* species among multiple photosynthetic pathways. **A** Results of PCA analysis, each point reflects the climatic information of each occurrence location. Red circles, green triangles and blue squares represent C_3_-CAM, C_3_ and CAM species, respectively. The levels of association between photosynthetic pathways and climatic factors were depicted using various colors; **B** Distribution of 11 *Dendrobium* species. Red, green and blue circles represent C_3_-CAM, C_3_ and CAM species analyzed in this study, respectively

A total of 144 distribution records of the eleven *Dendrobium* species were collected. On average, 13 records of each species were obtained from botanical histories and related literature [40, 41]. We marked the representative spots for each species on the map of China and discovered that the distribution of *Dendrobium* species with various photosynthetic routes was varied, e.g., CAM plants had the narrowest distribution range only distributed in Yunnan Province. C_3_ plants were mainly distributed in Guangxi, Hainan and Yunnan. While, C_3_-CAM plants had the widest distribution, indicating that the geographical distribution of *Dendrobium* species was related to the efficiency of photosynthetic pathways (Fig. 5B).

To further determine which bioclimatic factors are the main factor that contributed to the correlated relationship between geographical distribution and photosynthetic pathway, we performed PCA. PCA test the role of climate in determining different photosynthetic pathways by using the 2,736 bioclimatic factors of 11 *Dendrobium* species. The plot revealed that 144 bioclimatic points from 11 species were separated into three distinct groups (named groups 1–3, Fig. 5A). The three groups included points from group 1 (red circles), group 2 (green triangles) and group 3 (blue squares), which represented species in C_3_-CAM plants, C_3_ plants and CAM plants, respectively. It appears that the distribution of C_3_-CAM plants was more extensive than those in CAM plants and C_3_ plants, indicating that multiple photosynthetic pathways may be associated with a variety of factors in *Dendrobium*. According to PCA1, which indicated distinct differences in bio14 (precipitation of driest month), bio15 (precipitation of seasonality), bio17 (precipitation of driest quarter of the year) and bio19 (precipitation of coldest quarter of the year), C_3_-CAM plants were distinguished from the other groups (Fig. 5A). These findings suggested that precipitation, particularly the fluctuating ranges of precipitation, was the primary factor in the environmental differences between C_3_-CAM and other groups. PCA2 was the second component in the PCA analysis and separated C_3_ plants from the other groups (Fig. 5A). PCA2 could be predominantly characterized by the environmental factors of bio1(annual mean temperature), bio5 (max temperature of warmest month), bio6 (min temperature of coldest month), bio9 (mean temperature of driest quarter of the year), bio10 (mean temperature of warmest quarter of the year) and bio11 (mean temperature of coldest quarter of the year) (Fig. 5A), demonstrating that the main differences between group C_3_ and other species are associated to the changeable temperature ranges.

## Discussion

### Ensure the photosynthetic pathways could provide vital information for the study of CAM evolution

CAM, a specialized mode of the photosynthetic pathway, is an important adaptive feature of plants in drought or high-temperature conditions [42–44]. CAM pathway has evolved multiple times from C_3_ ancestors [4], for example, phylogenetics of the orchid family confirmed that CAM pathway may have evolved at least four times [45]. Moreover, it has become increasingly evident that the origin of CAM pathway may vary within orchid genera, especially in *Dendrobium* orchids [13]. However, because of the absence of a determination of photosynthetic traits, the origin of CAM pathway in *Dendrobium* orchids remains unknown. Recently, whole-tissue carbon isotope ratios (δ^13^C) have been used to categorize species as predominantly C_3_ or CAM [13]. However, carbon isotope analysis cannot identify species in which CAM was present but did not significantly impact overall carbon gain relative to C_3_. Therefore, we measured P_n_ to distinguish multiple photosynthetic pathways in *Dendrobium* more precisely. Based on the result of P_n_, we confirmed that there were various photosynthetic pathways, including C_3_, CAM and C_3_-CAM, among *Dendrobium* species. For example, the P_n_ in C_3_ plants was expanded to zero during daytime but below night, while below zero during the day but enhanced that the night in CAM plants (Supplementary Fig. 1). Based on our results, the selected 11 *Dendrobium* species were separated into three pathways. Combined with the comparative chloroplast genomic analysis and climatic correlation test, we believe that our findings could offer new insights into the evolution of CAM photosynthesis.

### Disproportional evolution of* Dendrobium* chloroplast genomes among different photosynthetic pathways

The evolution of CAM within the *Dendrobium* was mainly due to the high diversity of habitats inhabited by species in this genus [46–48]. To understand the origin of multiple photosynthetic pathways, we analyzed the photosynthetic characteristics and constructed the phylogenetic trees of 11 *Dendrobium* species. Indeed, based on our measurement of net photosynthetic rates and phylogenetic results, we suggested that CAM pathway has independently arisen at least two times among the 11 *Dendrobium* species. Additionally, we also evaluated the divergence time, showing that two CAM clades diverged at 12.07 and 11.31 mya (Fig. 2A), indicating rapid evolution to adapt to the environment.

Considering the diversified genome structure variations and the evolution patterns of protein-coding genes among *Dendrobium* species [45, 49, 50], we proposed that the evolution of the chloroplast genomes was disproportional among different photosynthetic pathways due to three reasons. Firstly, comparative research demonstrated significant variations in overall chloroplast genome characteristics of 11 *Dendrobium* species in two aspects, comprising basic chloroplast genome characteristics, especially the flanking IR/SC junctions (Supplementary Fig. 2). Secondly, the evolution of InDels among different photosynthetic pathways was inconsistent. For example, (i) the evolution of InDels differed in the different photosynthetic pathways (Fig. 3A). (ii) the occurrence rates and distribution densities of insertions and deletions among different photosynthetic pathways were asymmetrical. The distribution densities of InDels in LSC (insertion:35.14–37.84 bp/kbp; deletion: 45.14–58.26 bp/kbp), IR (insertion:24.76–26.57 bp/kbp; deletion: 6.68–8.55 bp/kbp) and SSC (insertion: 218.55–231.08 bp/kbp; deletion: 48.05–56.23 bp/kbp) regions demonstrated that the distribution of InDels was determined by their positions in chloroplast genomes (Supplementary Fig. 3). Thirdly, the evolution patterns of chloroplast genes among different photosynthetic pathways were diversified. (i) The chloroplast genomes of multiple photosynthetic pathways revealed various substitution rates. In this study, the value of dn and ds in CAM was higher than in C_3_-CAM and C_3_ species, indicating that the protein sequences of multiple photosynthetic pathways exhibited diverse evolution (Fig. 4A). (ii) The substitution rates of protein-coding genes among multiple photosynthetic pathways were inconsistent. For example, *atpI*, *ccsA* and *rps15* revealed the highest dn rates in the CAM category but the lowest in the C_3_ category. However, the dn rates of *petA* and *rps14* demonstrated the opposite result (Supplementary Table 2). (iii) Different clades exhibited various evolution patterns of adaptive genes. In 11 *Dendrobium* species, a total of 24 positively selective genes, e.g., *psbI*, *psbJ* and *psbL*, existed in different branches (Fig. 4B), suggesting that various photosynthetic pathways may have been crucial in the adaptive evolution of *Dendrobium*. Therefore, we concluded that multiple photosynthetic pathways contributed to the disproportional evolution of chloroplast genomes in *Dendrobium*.

### Temperature and precipitation influenced the evolution of photosynthetic pathways and promoted the establishment of CAM in *Dendrobium*

The complicated environmental changes, e.g., CO_2_ concentration and the decrease of water, have led to the rapid evolution of *Dendrobium* species, resulting in their various photosynthetic pathways, e.g., C_3_ pathway, C_3_-CAM and CAM pathway [20]. Recent studies have indicated that the photosynthetic pathways of *Dendrobium* species are closely related to their geographical distribution [13, 51]. In this study, 11 *Dendrobium* species were mainly located in southern China along the Qinling Mountains-Huaihe River border. Among these, CAM plants were only distributed in Yunnan province, while C_3_ plants were distributed in the provinces of Yunnan, Guangdong, and Hainan, all of which are located south of China’s Qinling-Huaihe River. C_3_-CAM plants have the broadest distribution and are found in more than ten provinces, mainly in Guangxi, Yunnan and Hainan provinces. Considering the relationship between their photosynthetic adaptive ability and their distribution ranges, e.g., C_3_-CAM *Dendrobium* orchids have broader distribution; however, C_3_ and CAM orchids contain narrower distribution (Fig. 5B), we proposed that the photosynthetic pathways of *Dendrobium* species are closely related to their geographical distribution.

Numerous factors, such as environmental changes [52, 53], colonization of dry environments [12] and rainfall seasonality [54–56], promote the evolution of photosynthesis. Especially, recent research indicated that moisture availability and temperature seasonality were confirmed as crucial factors in determining tropical woody plant evolution [57]. To identify the promoting factor of the evolution in *Dendrobium* species, we performed PCA (Fig. 5A), which separated into three groups corresponding to each group in different photosynthetic pathways. Based on the results of PCA (Fig. 5A), significant differences in bioclimatic factors such as annual mean temperature, max temperature of warmest month, and min temperature of coldest month were observed, indicating that environmental differences between C_3_-CAM and CAM were mainly linked to temperature. Meanwhile, the precipitation seasonality, e.g., precipitation of driest month, precipitation of seasonality, precipitation of driest quarter of the year and precipitation of coldest quarter of the year, was primarily responsible for the significant differences in environmental variables among multiple photosynthetic pathways. Therefore, we concluded that temperature and precipitation influenced the evolution of photosynthetic pathways and promoted the foundation of CAM.

## Supplementary Information

**Additional file 1: Supplementary Figure 1.** Diurnal change of net photosynthetic rate (P_n_) of the CAM, C_3_ and C_3_-CAM plants

**Additional file 2: Supplementary Figure 2.** IR/SC junction map of eleven *Dendrobium* orchids. Yellow represents the *rpl22* gene, blue represents the *ycf1* gene, red represents the *ndhF* gene and green represents the *psbA* gene.

**Additional file 3: Supplementary Figure 3.** InDels distribution densities in different regions of 11 *Dendrobium* orchids.

**Additional file 4: Supplementary Table 1.** The species information of 31 angiosperms used in the phylogenetic analysis

**Additional file 5: Supplementary Table 2.** dn and ds of 10 screened protein-coding genes in *Dendrobium.*

**Additional file 6: Supplementary Table 3.** The basic information of 10 screened protein-coding genes.

**Additional file 7: Supplementary Table 4.** The basic information of positively selective genes.

**Additional file 8: Supplementary Table 5.** Definition of nineteen bioclimatic factors

## Data Availability

All of the raw sequence reads used in this study have been deposited in NCBI (BioProject accession number: LC635345, LC635347, LC635346, LC635348, LC636120, LC636121, LC636124, LC636122, LC636123).
