# Supplementary figures and images for: The climate changes promoted the chloroplast genomic evolution of Dendrobium orchids among multiple photosynthetic pathways
Source: BMC Plant Biol. 2023 Apr 10;23:189. doi: 10.1186/s12870-023-04186-y (PMC10084689; doi:10.1186/s12870-023-04186-y)

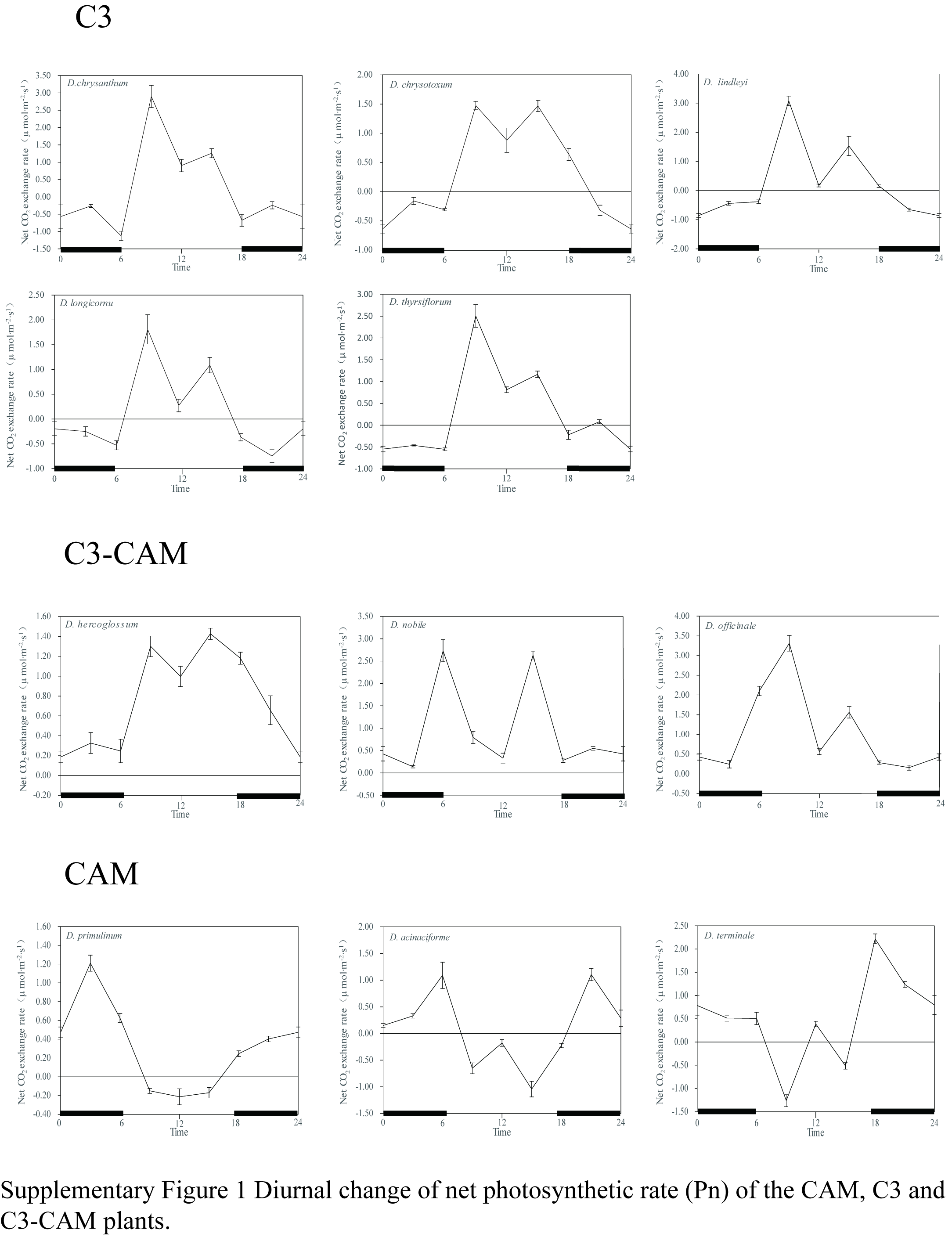

Supplement: Supplementary file 1 — Additional file 1: Supplementary Figure 1. Diurnal change of net photosynthetic rate (Pn) of the CAM, C3 and C3-CAM plants [file 12870_2023_4186_MOESM1_ESM.tif]

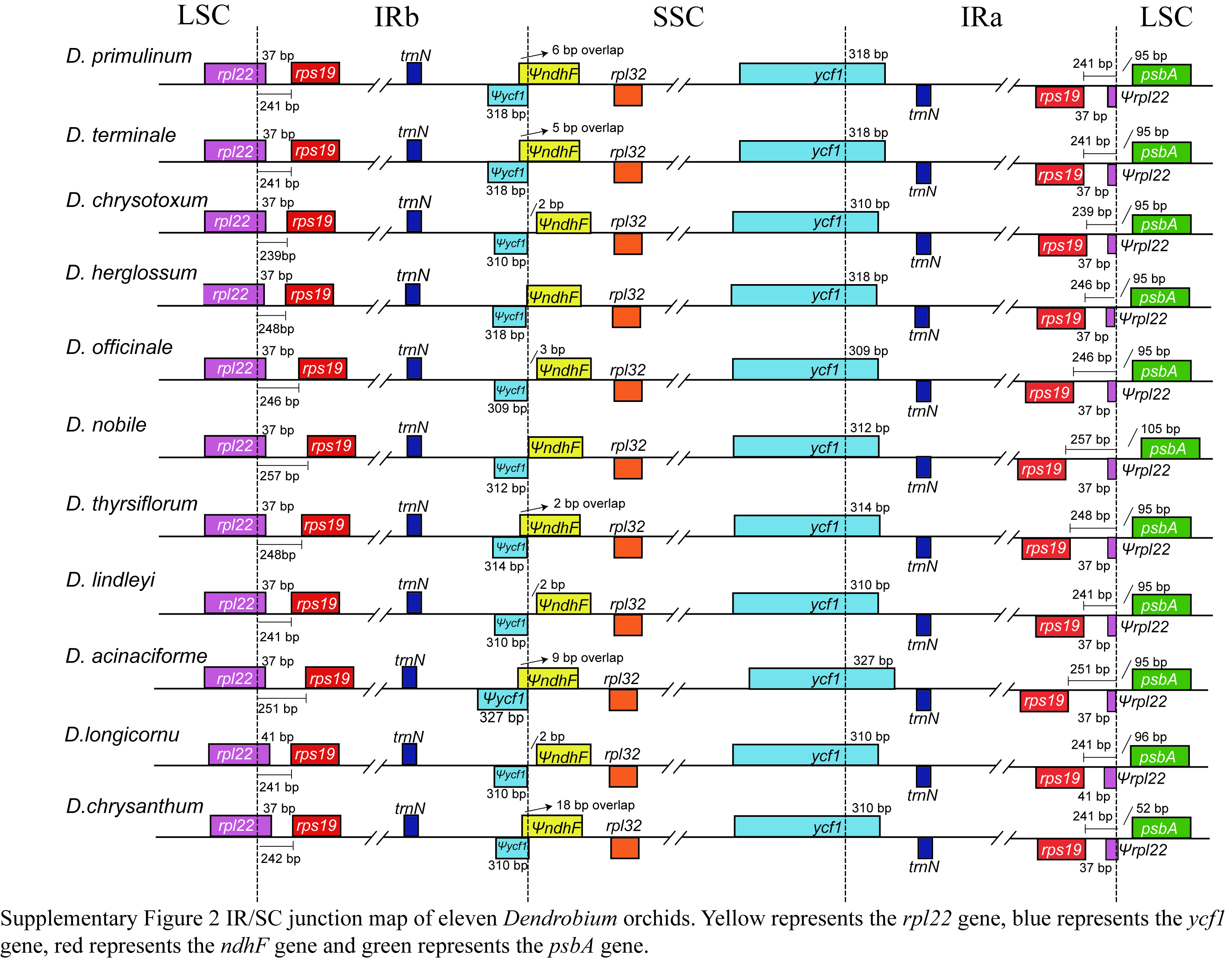

Supplement: Supplementary file 2 — Additional file 2: Supplementary Figure 2. IR/SC junction map of eleven Dendrobium orchids. Yellow represents the rpl22 gene, blue represents the ycf1 gene, red represents the ndhF gene and green represents the psbA gene. [file 12870_2023_4186_MOESM2_ESM.tif]

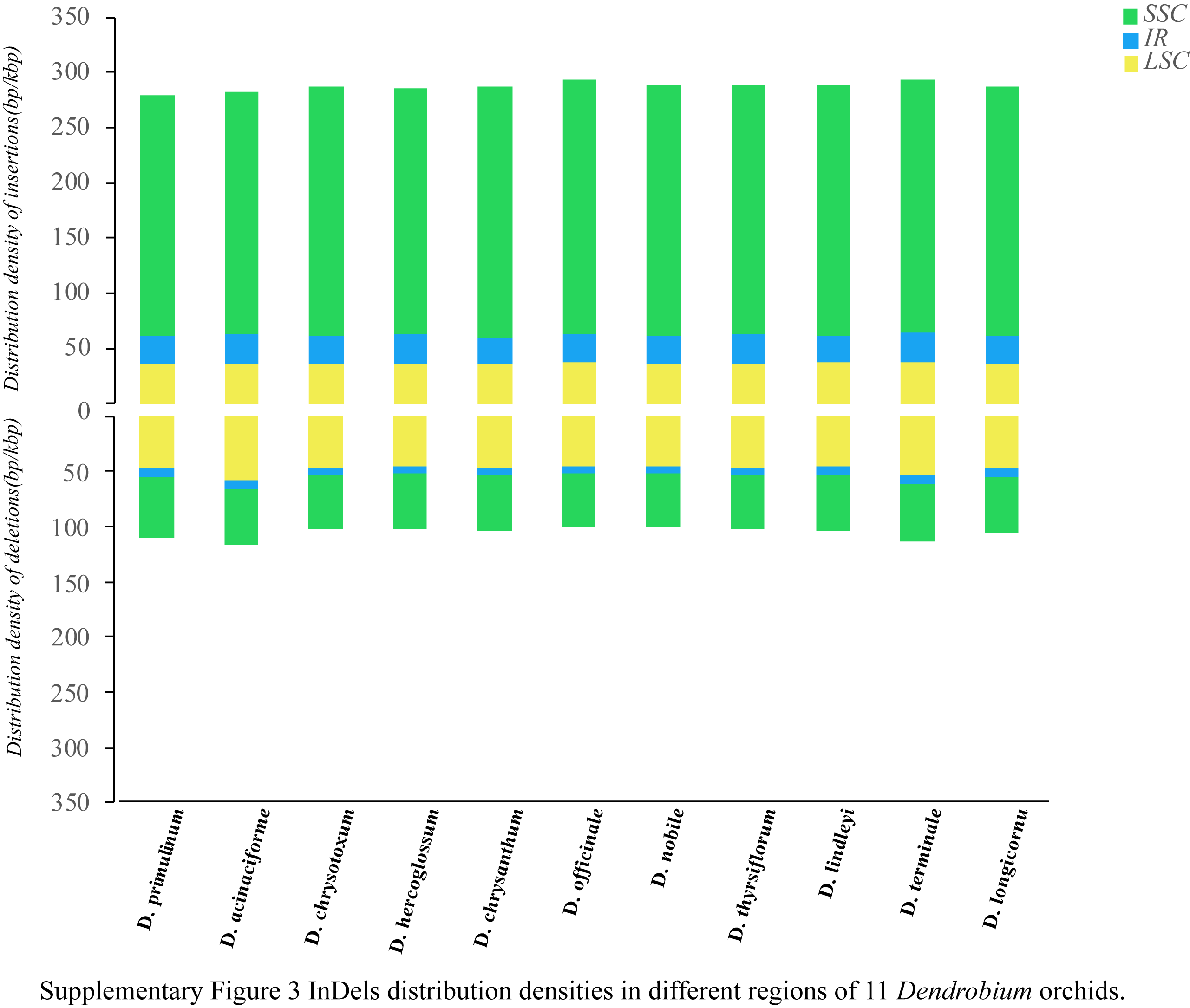

Supplement: Supplementary file 3 — Additional file 3: Supplementary Figure 3. InDels distribution densities in different regions of 11 Dendrobium orchids. [file 12870_2023_4186_MOESM3_ESM.tif]
