# Supplementary material for: The climate changes promoted the chloroplast genomic evolution of Dendrobium orchids among multiple photosynthetic pathways
Source: BMC Plant Biol. 2023 Apr 10;23:189. doi: 10.1186/s12870-023-04186-y (PMC10084689; doi:10.1186/s12870-023-04186-y)
Supplement: Supplementary file 4 — Additional file 4: Supplementary Table 1. The species information of 31 angiosperms used in the phylogenetic analysis [file 12870_2023_4186_MOESM4_ESM.docx]

| Class | Order | Family | Species | Accession no. |
| --- | --- | --- | --- | --- |
| Monocotyledoneae | Asparagales | Orchidaceae | *Goodyera fumata* | NC_026773 |
| Monocotyledoneae | Asparagales | Orchidaceae | *Goodyera schlechtendaliana* | LC085346 |
| Monocotyledoneae | Asparagales | Orchidaceae | *Goodyera procera* | NC_029363 |
| Monocotyledoneae | Asparagales | Orchidaceae | *Anoectochilus emeiensis* | NC_033895 |
| Monocotyledoneae | Asparagales | Orchidaceae | *Ludisia discolor* | NC_030540 |
| Monocotyledoneae | Asparagales | Orchidaceae | *Habenaria pantlingiana* | NC_026775 |
| Monocotyledoneae | Asparagales | Orchidaceae | *Dendrobium hercoglossum* | LC490400 |
| Monocotyledoneae | Asparagales | Orchidaceae | *Dendrobium primulinum* | LC635345 |
| Monocotyledoneae | Asparagales | Orchidaceae | *Dendrobium chrysanthum* | LC490683 |
| Monocotyledoneae | Asparagales | Orchidaceae | *Dendrobium terminale* | LC635346 |
| Monocotyledoneae | Asparagales | Orchidaceae | *Dendrobium lindleyi* | LC636121 |
| Monocotyledoneae | Asparagales | Orchidaceae | *Dendrobium thyrsiflorum* | LC636122 |
| Monocotyledoneae | Asparagales | Orchidaceae | *Dendrobium longicornu* | LC635347 |
| Monocotyledoneae | Asparagales | Orchidaceae | *Dendrobium nobile* | LC636120 |
| Monocotyledoneae | Asparagales | Orchidaceae | *Dendrobium officinale* | LC636123 |
| Monocotyledoneae | Asparagales | Orchidaceae | *Dendrobium chrysotoxum* | LC635348 |
| Monocotyledoneae | Asparagales | Orchidaceae | *Dendrobium acinaciforme* | LC636124 |
| Monocotyledoneae | Asparagales | Orchidaceae | *Dendrobium kingianum* | LC331062 |
| Monocotyledoneae | Asparagales | Orchidaceae | *Dendrobium salaccense* | NC_035332 |
| Monocotyledoneae | Asparagales | Orchidaceae | *Oncidium Gower Ramsey* | NC_014056 |
| Monocotyledoneae | Asparagales | Orchidaceae | *Oncidium sphacelatum* | NC_028148 |
| Monocotyledoneae | Asparagales | Orchidaceae | *Masdevallia coccinea* | NC_026541 |
| Monocotyledoneae | Asparagales | Orchidaceae | *Masdevallia picturata* | NC_026777 |
| Monocotyledoneae | Asparagales | Orchidaceae | *Cattleya crispata* | NC_026568 |
| Monocotyledoneae | Asparagales | Orchidaceae | *Calanthe triplicata* | NC_024544 |
| Monocotyledoneae | Asparagales | Orchidaceae | *Bletilla ochracea* | NC_029483 |
| Monocotyledoneae | Asparagales | Orchidaceae | *Bletilla striata* | NC_028422 |
| Monocotyledoneae | Asparagales | Orchidaceae | *Sobralia aff. bouchei* | NC_028209 |
| Monocotyledoneae | Asparagales | Orchidaceae | *Sobralia callosa* | NC_028147 |
| Monocotyledoneae | Asparagales | Orchidaceae | *Elleanthus sodiroi* | NC_027266 |
| Monocotyledoneae | Asparagales | Orchidaceae | *Bulbophyllum inconspicuum* | NC_046811 |

**Supplementary Table 1** The species information of 31 angiosperms used in the phylogenetic analysis
