# Supplementary material for: The climate changes promoted the chloroplast genomic evolution of Dendrobium orchids among multiple photosynthetic pathways
Source: BMC Plant Biol. 2023 Apr 10;23:189. doi: 10.1186/s12870-023-04186-y (PMC10084689; doi:10.1186/s12870-023-04186-y)
Supplement: Supplementary file 5 — Additional file 5: Supplementary Table 2. dn and ds of 10 screened protein-coding genes in Dendrobium. [file 12870_2023_4186_MOESM5_ESM.docx]

**Supplementary Table 2** dn and ds of 10 screened protein-coding genes in *Dendrobium*

| Species | Genes | dn | ds |
| --- | --- | --- | --- |
| *D. officinale* | *atpI* | 0.0107 | 0.067 |
|  | *ccsA* | 0.028 | 0.0399 |
|  | *cemA* | 0.0076 | 0.0281 |
|  | *clpP* | 0.0023 | 0.0222 |
|  | *matK* | 0.0405 | 0.0582 |
|  | *petA* | 0.0067 | 0.0537 |
|  | *rps14* | 0.0043 | 0.0292 |
|  | *rps15* | 0.0048 | 0.0509 |
|  | *rps3* | 0.0097 | 0.05 |
|  | *ycf1* | 0.0533 | 0.0709 |
| *D. chrysanthum* | *atpI* | 0.0107 | 0.0602 |
|  | *ccsA* | 0.0282 | 0.0289 |
|  | *cemA* | 0.0076 | 0.0281 |
|  | *clpP* | 0.0023 | 0.015 |
|  | *matK* | 0.0364 | 0.0624 |
|  | *petA* | 0.0067 | 0.059 |
|  | *rps14* | 0.0043 | 0.0158 |
|  | *rps15* | 0.0048 | 0.0509 |
|  | *rps3* | 0.0097 | 0.0322 |
|  | *ycf1* | 0.0505 | 0.071 |
| *D. thyrsiflorum* | *atpI* | 0.0125 | 0.0668 |
|  | *ccsA* | 0.0282 | 0.0242 |
|  | *cemA* | 0.013 | 0.0378 |
|  | *clpP* | 0.0023 | 0.015 |
|  | *matK* | 0.0374 | 0.0629 |
|  | *petA* | 0.0081 | 0.0698 |
|  | *rps14* | 0.0043 | 0.0158 |
|  | *rps15* | 0.0048 | 0.0509 |
|  | *rps3* | 0.0119 | 0.0465 |
|  | *ycf1* | 0.0497 | 0.0782 |
| *D.acinaciforme* | *atpI* | 0.0127 | 0.0925 |
|  | *ccsA* | 0.0309 | 0.0345 |
|  | *cemA* | 0.0139 | 0.0373 |
|  | *clpP* | 0.0023 | 0.015 |
|  | *matK* | 0.0401 | 0.0688 |
|  | *petA* | 0.0067 | 0.0893 |
|  | *rps14* | 0.0044 | 0.0287 |
|  | *rps15* | 0.014 | 0.064 |
|  | *rps3* | 0.0137 | 0.0402 |
|  | *ycf1* | 0.056 | 0.0891 |
| *D. lindleyi* | *atpI* | 0.0125 | 0.0607 |
|  | *ccsA* | 0.0279 | 0.03 |
|  | *cemA* | 0.0135 | 0.0403 |
|  | *clpP* | 0.0046 | 0.0145 |
|  | *matK* | 0.0374 | 0.0744 |
|  | *petA* | 0.0068 | 0.0572 |
|  | *rps14* | 0.0043 | 0.0158 |
|  | *rps15* | 0.0048 | 0.0509 |
|  | *rps3* | 0.0116 | 0.0409 |
|  | *ycf1* | 0.054 | 0.0887 |
| *D. nobile* | *atpI* | 0.0107 | 0.0602 |
|  | *ccsA* | 0.027 | 0.0334 |
|  | *cemA* | 0.0074 | 0.0384 |
|  | *clpP* | 0.0023 | 0.0222 |
|  | *matK* | 0.0397 | 0.0569 |
|  | *petA* | 0.0067 | 0.0537 |
|  | *rps14* | 0.0043 | 0.0292 |
|  | *rps15* | 0.0941 | 0.0509 |
|  | *rps3* | 0.0115 | 0.0432 |
|  | *ycf1* | 0.0511 | 0.069 |
| *D. chrysotoxum* | *atpI* | 0.0107 | 0.0602 |
|  | *ccsA* | 0.0269 | 0.0238 |
|  | *cemA* | 0.0074 | 0.0381 |
|  | *clpP* | 0.0023 | 0.015 |
|  | *matK* | 0.0352 | 0.0682 |
|  | *petA* | 0.0054 | 0.0529 |
|  | *rps14* | 0.0089 | 0.0271 |
|  | *rps15* | 0.0048 | 0.0509 |
|  | *rps3* | 0.0095 | 0.0344 |
|  | *ycf1* | 0.048 | 0.0806 |
| *D. terminale* | *atpI* | 0.0126 | 0.0939 |
|  | *ccsA* | 0.0307 | 0.0302 |
|  | *cemA* | 0.0116 | 0.0332 |
|  | *clpP* | 0.0023 | 0.0219 |
|  | *matK* | 0.0412 | 0.0685 |
|  | *petA* | 0.0067 | 0.0656 |
|  | *rps14* | 0.0044 | 0.0287 |
|  | *rps15* | 0.0138 | 0.0986 |
|  | *rps3* | 0.0115 | 0.0253 |
|  | *ycf1* | 0.057 | 0.0847 |
| *D. longicornu* | *atpI* | 0.0125 | 0.0607 |
|  | *ccsA* | 0.0284 | 0.0336 |
|  | *cemA* | 0.011 | 0.0475 |
|  | *clpP* | 0.0023 | 0.0219 |
|  | *matK* | 0.0352 | 0.0593 |
|  | *petA* | 0.0053 | 0.0598 |
|  | *rps14* | 0.0131 | 0.0297 |
|  | *rps15* | 0.0094 | 0.0601 |
|  | *rps3* | 0.0098 | 0.0397 |
|  | *ycf1* | 0.0507 | 0.0774 |
| *D. hercoglossum* | *atpI* | 0.0108 | 0.0663 |
|  | *ccsA* | 0.027 | 0.0334 |
|  | *cemA* | 0.0074 | 0.0384 |
|  | *clpP* | 0.0023 | 0.0222 |
|  | *matK* | 0.0408 | 0.0604 |
|  | *petA* | 0.0067 | 0.0537 |
|  | *rps14* | 0.0044 | 0.0418 |
|  | *rps15* | 0.0048 | 0.0509 |
|  | *rps3* | 0.0115 | 0.0432 |
|  | *ycf1* | 0.053 | 0.0702 |
| *D. primulinum* | *atpI* | 0.0107 | 0.0668 |
|  | *ccsA* | 0.0277 | 0.0311 |
|  | *cemA* | 0.0119 | 0.0245 |
|  | *clpP* | 0.0023 | 0.015 |
|  | *matK* | 0.0396 | 0.0781 |
|  | *petA* | 0.0054 | 0.0581 |
|  | *rps14* | 0.0043 | 0.0158 |
|  | *rps15* | 0.0098 | 0.0462 |
|  | *rps3* | 0.0096 | 0.0247 |
|  | *ycf1* | 0.0535 | 0.0814 |
