# Supplementary material for: The climate changes promoted the chloroplast genomic evolution of Dendrobium orchids among multiple photosynthetic pathways
Source: BMC Plant Biol. 2023 Apr 10;23:189. doi: 10.1186/s12870-023-04186-y (PMC10084689; doi:10.1186/s12870-023-04186-y)
Supplement: Supplementary file 6 — Additional file 6: Supplementary Table 3. The basic information of 10 screened protein-coding genes. [file 12870_2023_4186_MOESM6_ESM.docx]

**Supplementary Table 3** The basic information of 10 screened protein-coding genes

| Genes | Region | Category for genes |
| --- | --- | --- |
| *atpI* | LSC | Photosynthesis genes |
| *ccsA* | SSC | Other genes |
| *cemA* | LSC | Other gene |
| *clpP* | LSC | Other gene |
| *matK* | LSC | Other gene |
| *petA* | LSC | Cytochrome b6/f complex |
| *rps14* | LSC | Self-replication genes |
| *rps15* | SSC | Self-replication genes |
| *rps3* | LSC | Self-replication genes |
| *ycf1* | SSC | Hypothetical chloroplast reading frames |
