# Supplementary material for: The climate changes promoted the chloroplast genomic evolution of Dendrobium orchids among multiple photosynthetic pathways
Source: BMC Plant Biol. 2023 Apr 10;23:189. doi: 10.1186/s12870-023-04186-y (PMC10084689; doi:10.1186/s12870-023-04186-y)
Supplement: Supplementary file 7 — Additional file 7: Supplementary Table 4. The basic information of positively selective genes. [file 12870_2023_4186_MOESM7_ESM.docx]

**Supplementary Table 4** The basic information of positively selective genes

| Branches | Genes | Regions | Category for genes |
| --- | --- | --- | --- |
| 1 | *psbI* | LSC | Photosystem II |
|  | *psbJ* | LSC | Photosystem II |
|  | *psbL* | LSC | Photosystem II |
|  | *rps7* | IR | Ribosomal proteins |
|  | *ycf2* | IR | Hypothetical chloroplast reading frames |
| 2 | *accD* | LSC | Other gene |
|  | *ccsA* | SSC | Other gene |
|  | *psaI* | LSC | PhotosystemⅠ |
|  | *psbF* | LSC | Photosystem II |
|  | *rps18* | LSC | Ribosomal proteins |
|  | *rps7* | IR | Ribosomal proteins |
| 3 | *atpE* | LSC | ATP synthase |
|  | *psbJ* | LSC | Photosystem II |
|  | *psbL* | LSC | Photosystem II |
|  | *ycf2* | IR | Hypothetical chloroplast reading frames |
| 4 | *accD* | LSC | Other gene |
|  | *infA* | LSC | Hypothetical chloroplast reading frames |
|  | *petG* | LSC | Cytochrome b6/f complex |
|  | *psbI* | LSC | Photosystem II |
|  | *rbcL* | LSC | RubisCO large subunit |
| 5 | *psbD* | LSC | Photosystem II |
|  | *psbL* | LSC | Photosystem II |
|  | *rps18* | LSC | Ribosomal proteins |
|  | *rps7* | IR | Ribosomal proteins |
