# Supplementary material for: The climate changes promoted the chloroplast genomic evolution of Dendrobium orchids among multiple photosynthetic pathways
Source: BMC Plant Biol. 2023 Apr 10;23:189. doi: 10.1186/s12870-023-04186-y (PMC10084689; doi:10.1186/s12870-023-04186-y)
Supplement: Supplementary file 8 — Additional file 8: Supplementary Table 5. Definition of nineteen bioclimatic factors [file 12870_2023_4186_MOESM8_ESM.docx]

**Supplementary Table 5** Definition of nineteen bioclimatic factors

| Code | Bioclimatic factors | Code | Bioclimatic factors |
| --- | --- | --- | --- |
| bio1 | annual mean temperature | bio11 | mean temperature of coldest quarter of the year |
| bio2 | mean diurnal range in temperature | bio12 | annual precipitation |
| bio3 | isothermality (monthly/annual temperature range) | bio13 | precipitation of wettest month |
| bio4 | temperature seasonality | bio14 | precipitation of driest month |
| bio5 | max temperature of warmest month | bio15 | precipitation of seasonality (coefficient of variation) |
| bio6 | min temperature of coldest month | bio16 | precipitation of wettest quarter of the year |
| bio7 | annual range in temperature | bio17 | precipitation of driest quarter of the year |
| bio8 | mean temperature of wettest quarter of the year | bio18 | precipitation of warmest quarter of the year |
| bio9 | mean temperature of driest quarter of the year | bio19 | precipitation of coldest quarter of the year |
| bio10 | mean temperature of warmest quarter of the year |  |  |

* The spatial resolution of the climate layers was 1 km^2^, and the climate data used to create the layers were restricted to records from 1970 to 2000.
